# Supplementary material for: Ephedrae Herba and Cinnamomi Cortex interactions with G glycoprotein inhibit respiratory syncytial virus infectivity
Source: Commun Biol. 2022 Jan 25;5:94. doi: 10.1038/s42003-022-03046-z (PMC8789818; doi:10.1038/s42003-022-03046-z)
Supplement: Supplementary file 2 — Supplementary Information [file 42003_2022_3046_MOESM2_ESM.pdf]

Supplementary Information for

Ephedrae Herba and Cinnamomi Cortex interactions with G glycoprotein inhibit respiratory syncytial virus infectivity

Table of Contents:

Supplementary Figures (Figs. 1-8)	pages 2-9
Supplementary Figures (Uncropped Western Blots)	pages 10-12
Supplementary Table	pages 13
Supplementary References	pages 14

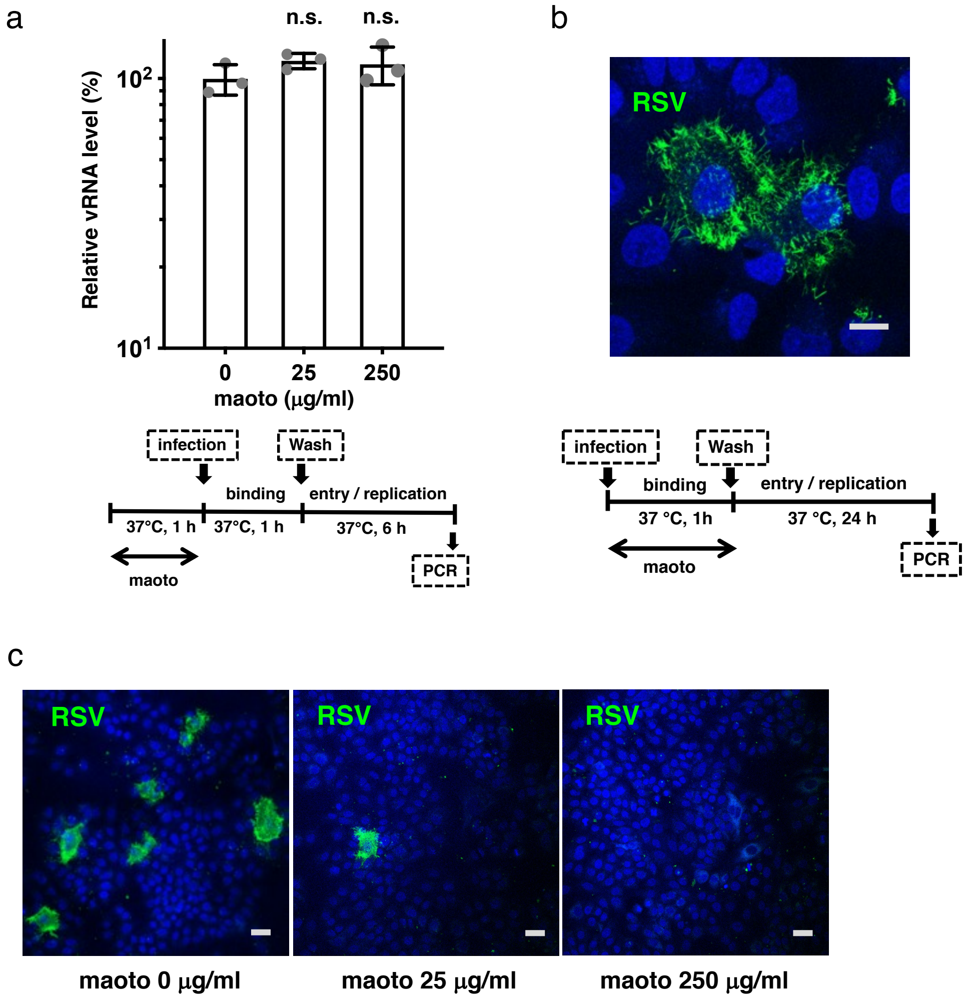

Supplementary Fig. 1. Anti-RSV activity of maoto in cultured cells. a Failure to elicit anti-RSV activity by pre-treatment of cells with maoto. A549 cells were treated with maoto for 1 hour and then washed-out prior to infection with RSV-A (moi 1). Viral RNA (vRNA) in cell extracts was quantified by RT-PCR. The culture conditions are illustrated below. The results show individual values and mean ± error bars SD (n=3). One-way ANOVA followed by Dunnett post-test. n.s.; not significant. **b** Intracellular localization of RSV G protein visualized by confocal microscopy. A549 cells were infected with RSV and cultured for 24 hours. Scale bar indicates 10 μm. **c** Reduced frequency of RSV-positive cells after treatment with maoto (moi 1). Photo images by confocal microscopy are shown. Scale bar indicates 10 μm. The culture conditions were the same as in **b**.

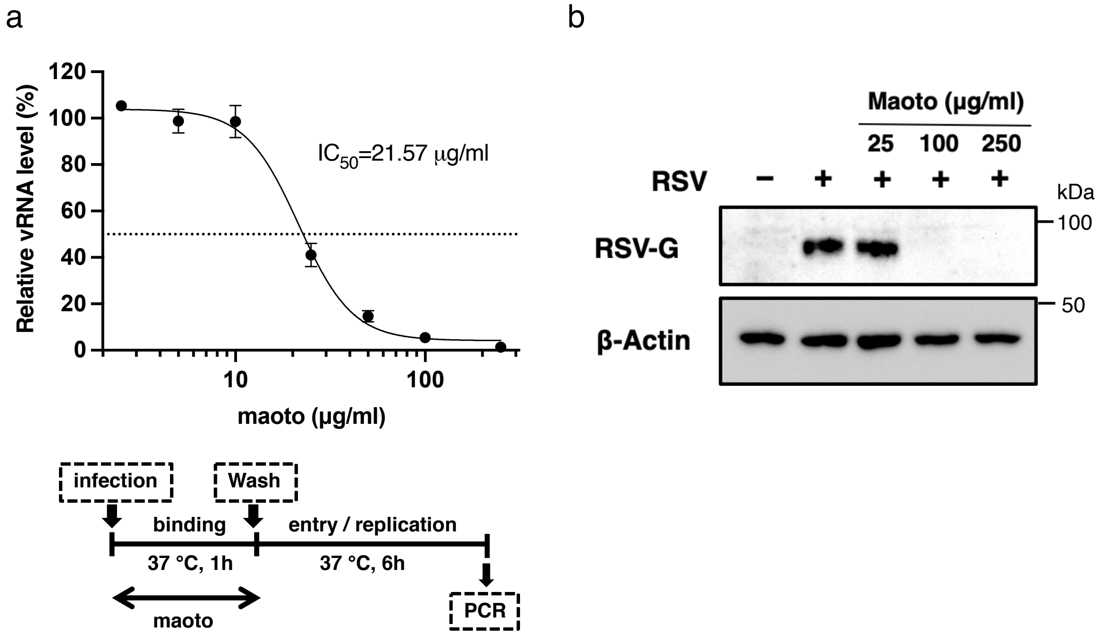

Supplementary Fig. 2. Anti-RSV activity of maoto in cultured cells at a moi of 10. a Dose-dependent decrease of vRNA by maoto. A549 cells were infected with RSV-A (moi 10) and treated with several dose of maoto at the binding phase. A measure of IC50 was determined using logistic regression testing. The culture conditions are illustrated bellow. The results show individual values and mean ± error bars SD (n=3). **b** Reduction of cell surface RSV G protein levels by maoto. A549 cells were inoculated with RSV (moi 10) and simultaneously treated with maoto on ice for 1 h. RSV G protein in the cells was detected by western blotting.

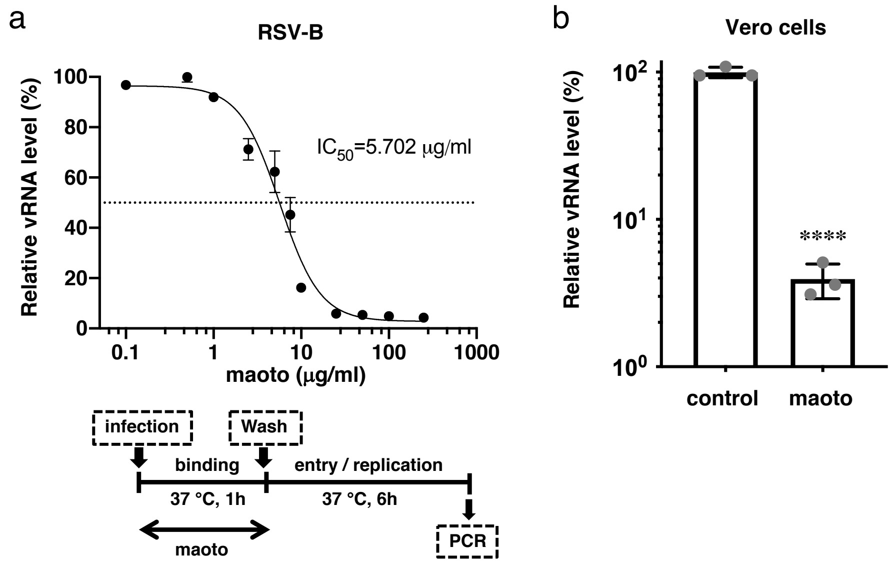

Supplementary Fig. 3. Maoto acts independently of both viral subtype and host cell line. a Antiviral effect of maoto in cells infected with RSV-B. A measure of IC50 was determined using logistic regression testing. The culture conditions were illustrated below. **b** Anti-RSV effect of maoto (25 *μ*g/ml) in Vero cells. The culture conditions were the same as in **a**. The results show individual values and mean ± error bars SD (n=3). Unpaired Student's *t*-test was used. **** $P < 0.0001$.

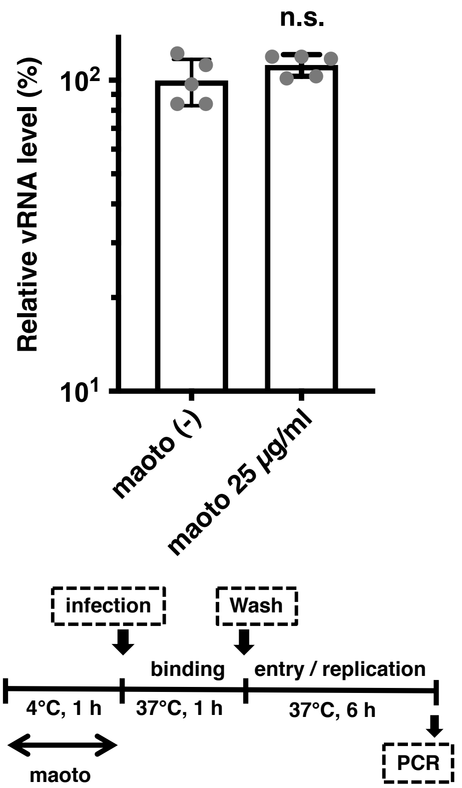

Supplementary Fig. 4. No effect of maoto on cellular receptors for RSV. Cells were pre-treated with maoto for 1 h prior to RSV infection on ice. After 6 h, viral RNA levels in cell lysates were measured by RT-PCR. The culture conditions are illustrated below. The results show individual values and mean ± error bars SD (n=5). Unpaired Student's *t*-test was used. n.s.; not significant.

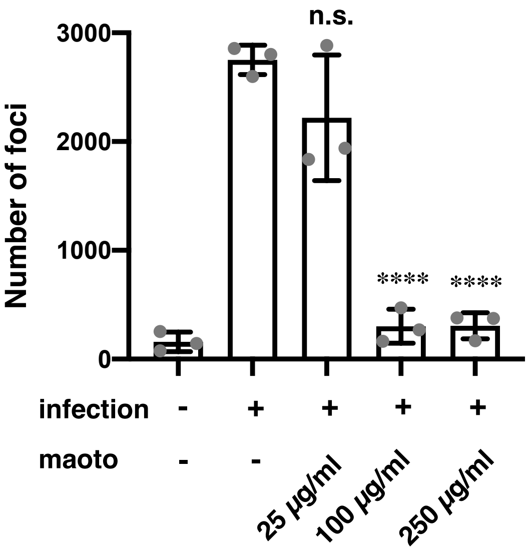

Supplementary Fig. 5. Reduction of cell surface RSV by maoto. A549 cells were inoculated with RSV (moi 10) and simultaneously treated with maoto on ice for 1 h. Numbers of RSV-positive foci on the cell surface observed by confocal microscopy were counted and plotted. The results show individual values and mean ± error bars SD (n=3). One-way ANOVA followed by Dunnett post-test . ****$P < 0.0001$ and n.s.; not significant.

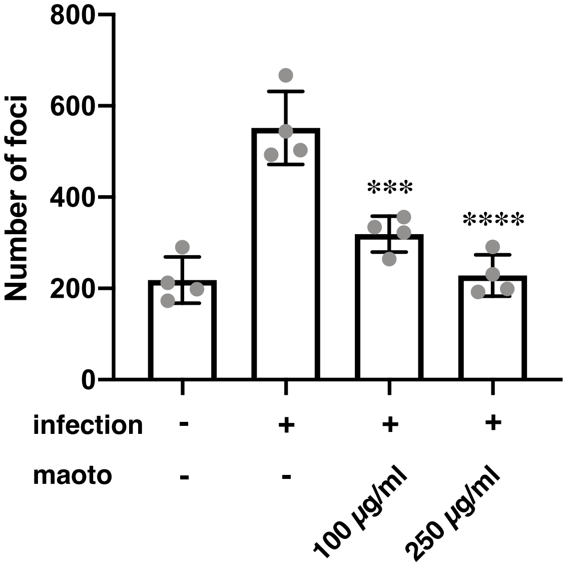

Supplementary Fig. 6. Inhibited interaction of G protein and CX3CR1 by maoto. A549 cells were attached with RSV (moi 10) for one hour on ice. The samples were incubated with primary antibodies against G protein and CX3CR1, followed by binding of the PLA probes. Circle DNA between the two probes was amplified with fluorescent-labeled oligonucleotides, and analyzed by the confocal microscopy. Mean foci were counted from 4 fields in each sample. The results show individual values and mean $\pm$ error bars SD (n=4). One-way ANOVA followed by Dunnett post-test. ***$P < 0.001$ and ****$P < 0.0001$.

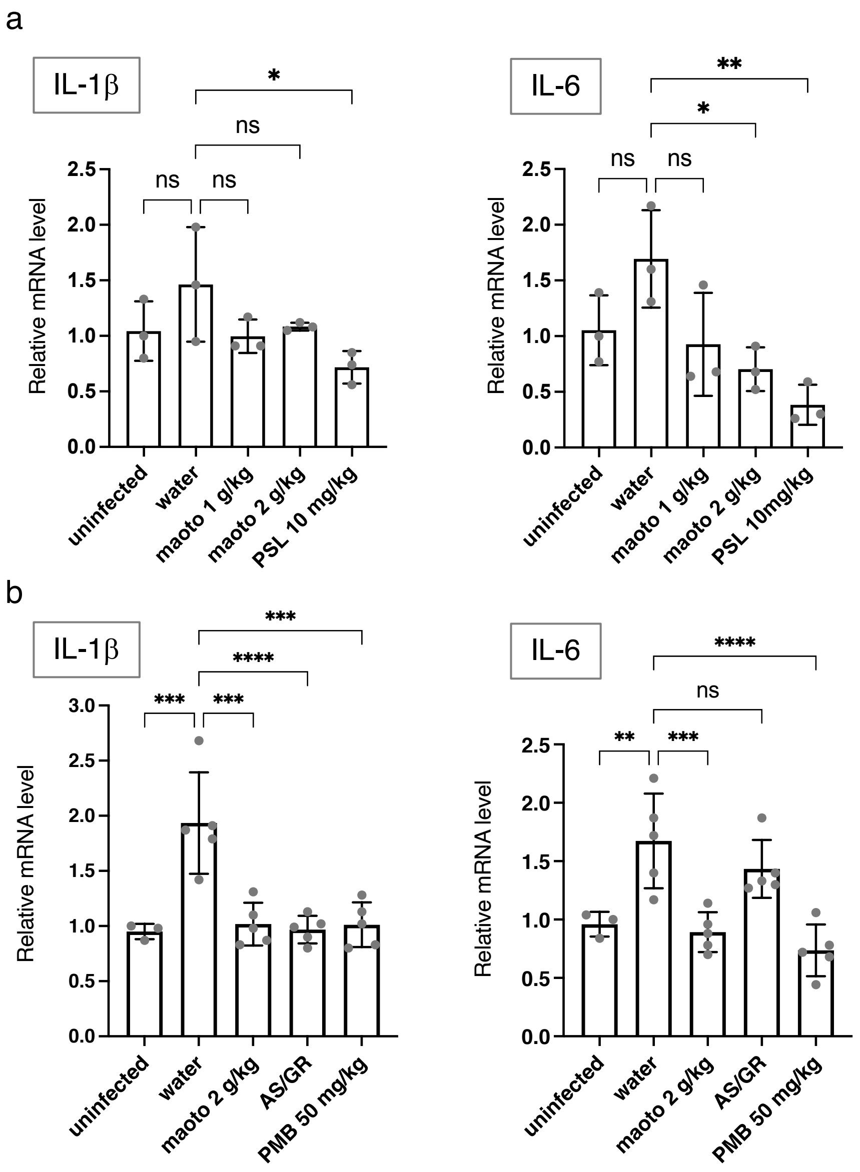

Supplementary Fig. 7. Cytokine production of mouse lung infected with RSV. a Experiments 1. **b** Experiment 2. **a** Female BALB/c mice were intranasally infected with RSV (1.8 × 10^6 PFU), and then orally administered maoto (1,000 mg/kg or 2,000 mg/kg), water (10 mL/kg), or prednisolone (10 mg/kg) for 5 days. IL-1β and IL-6 mRNA levels in lung tissues of mouse were measured by RT-PCR. **b** Female BALB/c mice were intranasally infected with RSV (1.8 × 10^6 PFU), and then orally administered maoto (2,000 mg/kg), water (10 mL/kg), AS/GR (645 mg/kg, 194 mg/kg, respectively), or PMB (50 mg/kg) for 5 days. IL-1β and IL-6 mRNA levels in lung tissues of mouse were measured by RT-PCR. The results show individual values and mean ± error bars SD (a: n=3, b: n=5). ANOVA followed by Dunnett post-test. *$P < 0.05$, **$P < 0.01$, ***$P < 0.001$, ****P<0.0001, and n.s.; not significant. AS: Armeniacae Semen, GR: Glycyrrhize Radix, PMB, palivizumab.

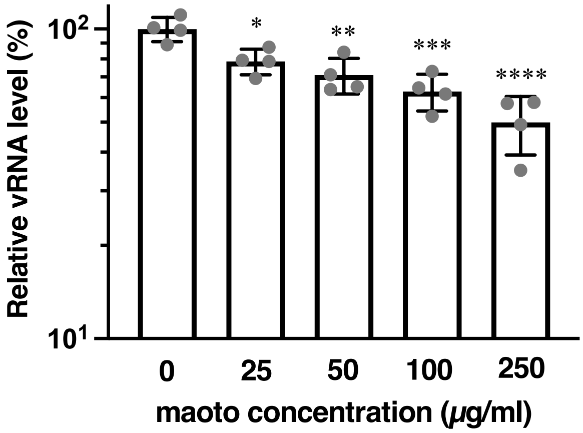

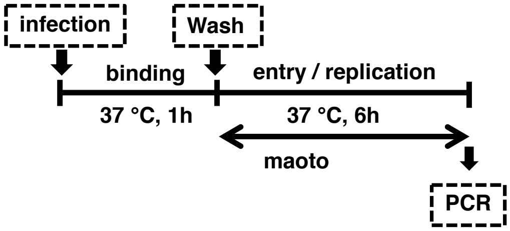

Supplementary Fig. 8. Increased concentration of maoto inhibits replication of virus in the entry/replication phase. After A549 cells were inoculated with RSV (moi 1) maoto was added in the entry/replication phase. After 6 h, viral RNA levels in cell lysates were measured by RT-PCR. The results show individual values and mean ± error bars SD (n=4). One-way ANOVA followed by Dunnett post-test. *$P < 0.05$, **$P < 0.01$, ***$P < 0.001$ and ****$P < 0.0001$.

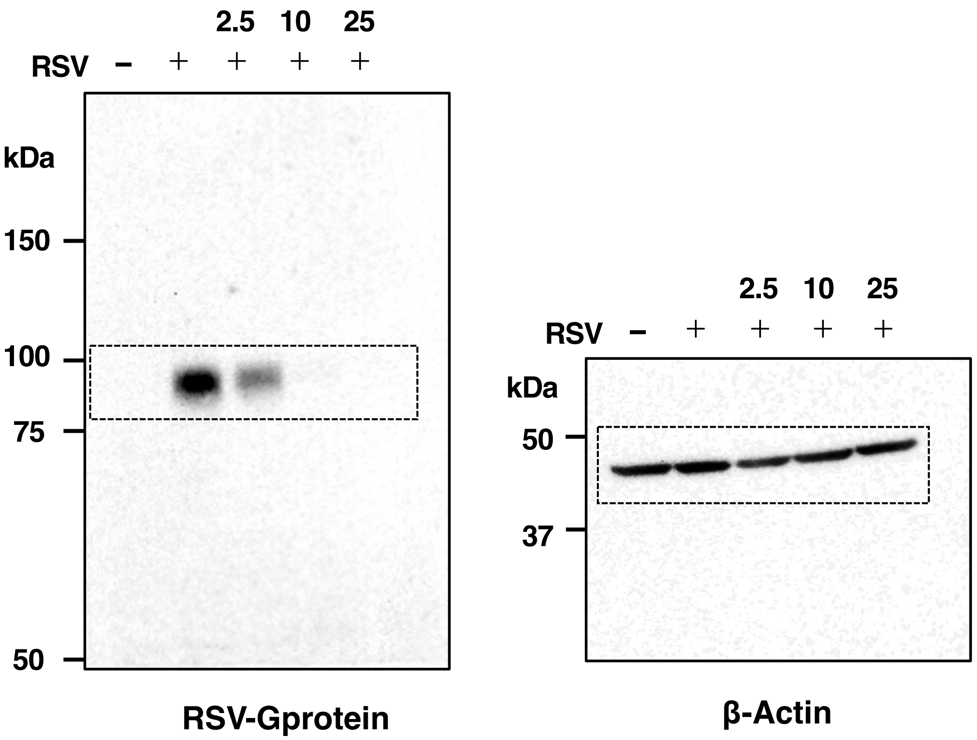

Supplementary Fig. 9. Uncropped Western Blots for Fig. 2d.

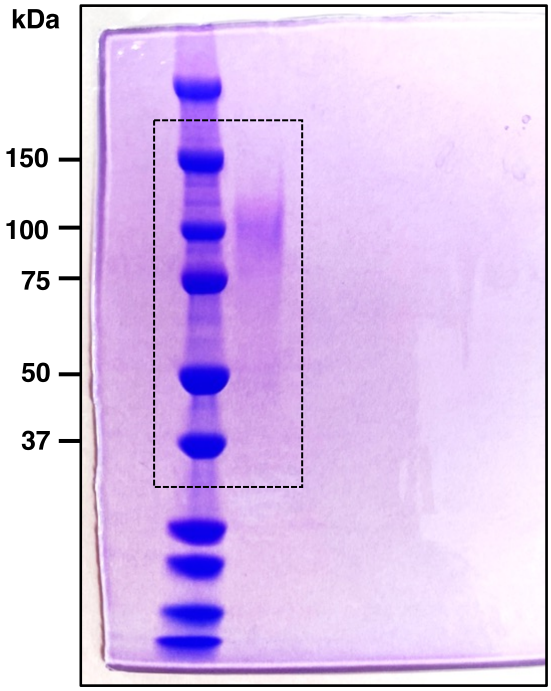

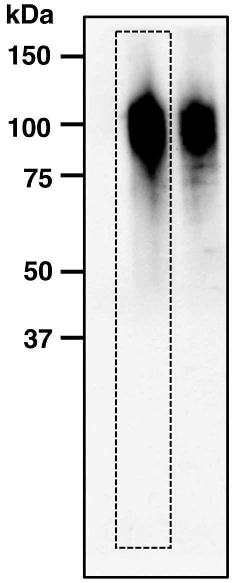

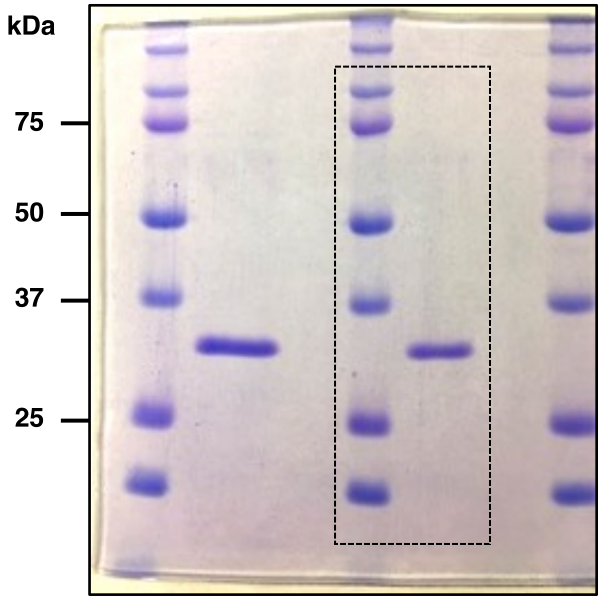

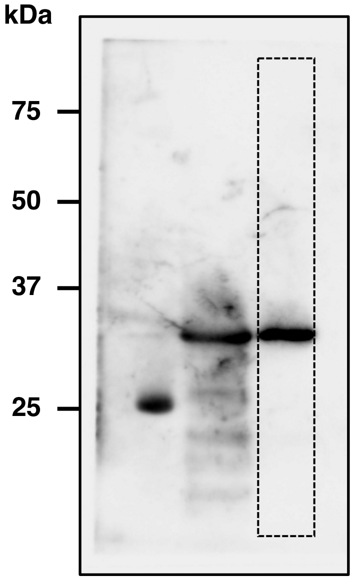

Supplementary Fig. 10. Uncropped Western Blots for Fig. 3d.

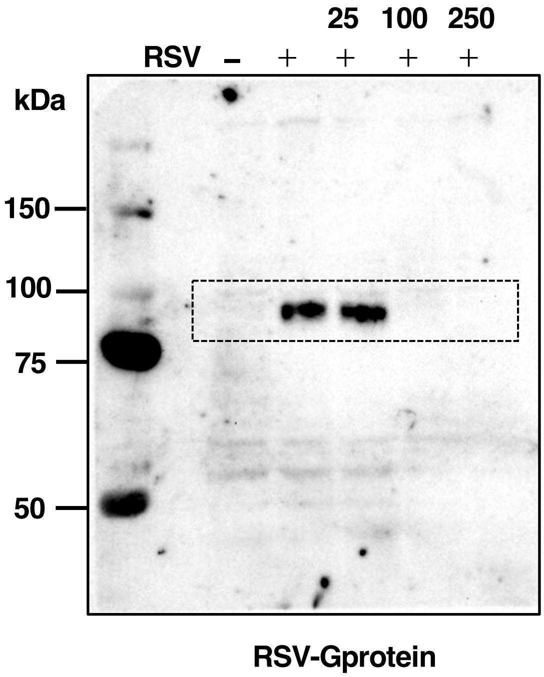

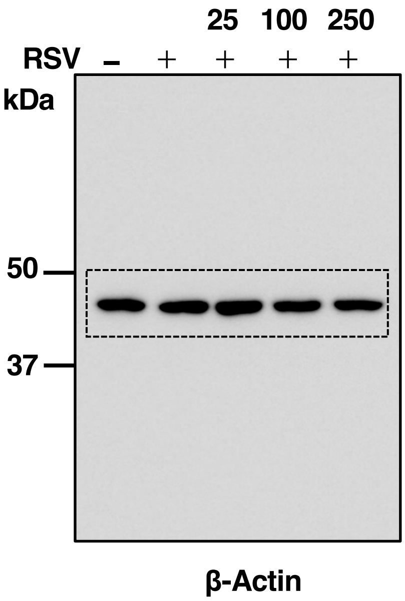

Supplementary Fig. 11. Uncropped Western Blots for Supplementary Fig. 2b.

Supplementary Table 1. Characteristics of maoto extract.

	Major components	Weghit ratio (%)*	References
Ephedrae Herba (EH)	ephedrine, pseudoephedrine, methylephedrine	32.3	1, 2
Cinnamomi Acid (CC)	cinnamaldehyde, procyanidin, cinnacassiol A	32.3	3, 4
Armeniacae Semen (AS)	amygdalin, mandelonitrile	25.8	5, 6
Glycyrrhize Radix (GR)	glycyrrhizic acid, liquiritin, isoliquiritin	9.6	7, 8, 9

* Percentages of dried weight of each plant before decoction by boiling in water.

Supplementary References

1. Dai, Y. *et al.* Quality marker identification based on standard decoction of differently processed materials of Ephedrae Herba. *J Ethnopharmacol* 237, 47-54, doi:10.1016/j.jep.2019.03.025 (2019).
2. Ibragic, S. & Sofić, E. Chemical composition of various Ephedra species. *Bosn J Basic Med Sci* 15, 21-27, doi:10.17305/bjbms.2015.539 (2015).
3. Lee, M. R. The history of Ephedra (ma-huang). *J R Coll Physicians Edinb* 41, 78-84, doi:10.4997/jrcpe.2011.116 (2011).
4. Wang, S. *et al.* The comparative pharmacokinetics of four bioactive ingredients after administration of Ramulus Cinnamomi-Radix Glycyrrhizae herb pair extract, Ramulus Cinnamomi extract and Radix Glycyrrhizae extract. *Biomed Chromatogr* 30, 1270-1277, doi:10.1002/bmc.3677 (2016).
5. Wu, X., He, J., Xu, H., Bi, K. & Li, Q. Quality assessment of Cinnamomi Ramulus by the simultaneous analysis of multiple active components using high-performance thin-layer chromatography and high-performance liquid chromatography. *J Sep Sci* 37, 2490-2498, doi:10.1002/jssc.201400494 (2014).
6. Moertel, C. G. *et al.* A clinical trial of amygdalin (Laetrile) in the treatment of human cancer. *N Engl J Med* 306, 201-206, doi:10.1056/NEJM198201283060403 (1982).
7. Song, S. *et al.* Stereoselective metabolism of amygdalin-based study of detoxification of Semen Armeniacae Amarum in the Herba Ephedrae-Semen Armeniacae Amarum herb pair. *J Ethnopharmacol* 179, 356-366, doi:10.1016/j.jep.2015.12.019 (2016).
8. Pastorino, G., Cornara, L., Soares, S., Rodrigues, F. & Oliveira, M. B. P. P. Liquorice (Glycyrrhiza glabra): A phytochemical and pharmacological review. *Phytother Res* 32, 2323-2339, doi:10.1002/ptr.6178 (2018).
9. Yang, R., Wang, L. Q., Yuan, B. C. & Liu, Y. The Pharmacological Activities of Licorice. *Planta Med* 81, 1654-1669, doi:10.1055/s-0035-1557893 (2015).
