# Supplementary material for: Ephedrae Herba and Cinnamomi Cortex interactions with G glycoprotein inhibit respiratory syncytial virus infectivity
Source: Commun Biol. 2022 Jan 25;5:94. doi: 10.1038/s42003-022-03046-z (PMC8789818; doi:10.1038/s42003-022-03046-z)
Supplement: Supplementary file 3 — Description of Additional Supplementary Files [file 42003_2022_3046_MOESM3_ESM.pdf]

Description of Additional Supplementary Files

File name: Supplementary Data 1

Description: Original data for main figures. Source data for real-time PCR, plaque-forming unit assay, and surface plasmon resonance.

File name: Supplementary Data 2

Description: Original data for supplementary figures. Source data for real-time PCR and the quntification of virion foci by immunofluorescence microscopy.
