# Supplementary material for: Ephedrae Herba and Cinnamomi Cortex interactions with G glycoprotein inhibit respiratory syncytial virus infectivity
Source: Commun Biol. 2022 Jan 25;5:94. doi: 10.1038/s42003-022-03046-z (PMC8789818; doi:10.1038/s42003-022-03046-z)
Supplement: Supplementary file 6 — Reporting Summary [file 42003_2022_3046_MOESM6_ESM.pdf]

nature research

Corresponding author(s): Shigeki Nabeshima

Last updated by author(s): Dec 15, 2021

Reporting Summary

Nature Research wishes to improve the reproducibility of the work that we publish. This form provides structure for consistency and transparency in reporting. For further information on Nature Research policies, see our Editorial Policies and the Editorial Policy Checklist.

Statistics

For all statistical analyses, confirm that the following items are present in the figure legend, table legend, main text, or Methods section.

n/a	Confirmed	
☐	☒	The exact sample size (*n*) for each experimental group/condition, given as a discrete number and unit of measurement
☐	☒	A statement on whether measurements were taken from distinct samples or whether the same sample was measured repeatedly
☐	☒	The statistical test(s) used AND whether they are one- or two-sided<br>*Only common tests should be described solely by name; describe more complex techniques in the Methods section.*
☐	☒	A description of all covariates tested
☐	☒	A description of any assumptions or corrections, such as tests of normality and adjustment for multiple comparisons
☐	☒	A full description of the statistical parameters including central tendency (e.g. means) or other basic estimates (e.g. regression coefficient) AND variation (e.g. standard deviation) or associated estimates of uncertainty (e.g. confidence intervals)
☐	☒	For null hypothesis testing, the test statistic (e.g. *F*, *t*, *r*) with confidence intervals, effect sizes, degrees of freedom and *P* value noted<br>*Give P values as exact values whenever suitable.*
☒	☐	For Bayesian analysis, information on the choice of priors and Markov chain Monte Carlo settings
☒	☐	For hierarchical and complex designs, identification of the appropriate level for tests and full reporting of outcomes
☒	☐	Estimates of effect sizes (e.g. Cohen's *d*, Pearson's *r*), indicating how they were calculated

Our web collection on statistics for biologists contains articles on many of the points above.

Software and code

Policy information about availability of computer code

Data collection	Data was not collected using any code in this study.
Data analysis	CFX 3.1 software (Bio-Rad), ImageJ software (Wayne Rasband), BIAevaluation software (GE Healthcare), GraphPad Prism software (GraphPad Software), and Microsoft Excel.

For manuscripts utilizing custom algorithms or software that are central to the research but not yet described in published literature, software must be made available to editors and reviewers. We strongly encourage code deposition in a community repository (e.g. GitHub). See the Nature Research guidelines for submitting code & software for further information.

Data

Policy information about availability of data

All manuscripts must include a data availability statement. This statement should provide the following information, where applicable:

- Accession codes, unique identifiers, or web links for publicly available datasets
- A list of figures that have associated raw data
- A description of any restrictions on data availability

All the processed data are available within the article. Raw data in the main figures and the supplementary informeations are provided in the Supplementary data file (two Excel files).

Field-specific reporting

Please select the one below that is the best fit for your research. If you are not sure, read the appropriate sections before making your selection.

☒ Life sciences ☐ Behavioural & social sciences ☐ Ecological, evolutionary & environmental sciences

For a reference copy of the document with all sections, see nature.com/documents/nr-reporting-summary-flat.pdf

Life sciences study design

All studies must disclose on these points even when the disclosure is negative.

Sample size	The number of samples was determined to be adequate based on the degree and consistency of differences between groups.
Data exclusions	No data were excluded from analysis.
Replication	All experiments were performed with inter-experimental replicates. Number of replicates is defined in the figure legends.
Randomization	Randomization is not relevant to our study design.
Blinding	Blinding was not necessary in this study.

Reporting for specific materials, systems and methods

We require information from authors about some types of materials, experimental systems and methods used in many studies. Here, indicate whether each material, system or method listed is relevant to your study. If you are not sure if a list item applies to your research, read the appropriate section before selecting a response.

Materials & experimental systems

n/a	Involved in the study	
☐	☒	Antibodies
☐	☒	Eukaryotic cell lines
☒	☐	Palaeontology and archaeology
☐	☒	Animals and other organisms
☒	☐	Human research participants
☒	☐	Clinical data
☒	☐	Dual use research of concern

Methods

n/a	Involved in the study	
☒	☐	ChIP-seq
☒	☐	Flow cytometry
☒	☐	MRI-based neuroimaging

Antibodies

Antibodies used	Human RSV G protein (94966; Abcam Cambridge, UK), β-actin (A5316; Sigma-Aldrich), CX3CR1 (8021; Abcam), and anti-mouse antibody conjugated with Alexa Fluor 488 (A11029; ThermoFisher).
Validation	All antibodies used have been validaded by the supplier for use in Western blotting and /or fluoresecent microscopy.

Eukaryotic cell lines

Policy information about cell lines

Cell line source(s)	A549 cell line from lab stock originally purchased from ATCC (CCL-185).
Authentication	n/a
Mycoplasma contamination	We confirm that A549 cell line tested negative for mycoplasma contamination.
Commonly misidentified lines (See ICLAC register)	n/a

Animals and other organisms

Policy information about studies involving animals; ARRIVE guidelines recommended for reporting animal research

Laboratory animals	Female BALB/c mice were purchased from SLC (Hamamatsu, Shizuoka, Japan), and used for the experiment at 6 weeks old.

Wild animals	n/a
Field-collected samples	n/a
Ethics oversight	All animal experiments were approved by the Laboratory Animal Committee of Nihon Bioresearch (Hashima, Gifu, Japan) and Tsumura & Co (Tokyo, Japan), and performed in accordance with guidelines for the conduct of animal experiments in ministry of health, labour and welfare, Japan.

Note that full information on the approval of the study protocol must also be provided in the manuscript.
